# Impact of Lysine Succinylation on the Biology of Fungi

**DOI:** 10.3390/cimb46020065

**Published:** 2024-01-23

**Authors:** John Adejor, Elisabeth Tumukunde, Guoqi Li, Hong Lin, Rui Xie, Shihua Wang

**Affiliations:** Key Laboratory of Pathogenic Fungi and Mycotoxins of Fujian Province, Key Laboratory of Biopesticide and Chemical Biology of Education Ministry, College of Life Sciences, Fujian Agriculture and Forestry University, Fuzhou 350002, China; johnadejor63@gmail.com (J.A.); elizawkb22@gmail.com (E.T.); liguoqi0927@163.com (G.L.); linhong2805@163.com (H.L.); xr37154@163.com (R.X.)

**Keywords:** aflatoxin, fungi, post-translational modification, protein lysine acylation, SIRT5, succinyl-CoA, succinyltransferase

## Abstract

Post-translational modifications (PTMs) play a crucial role in protein functionality and the control of various cellular processes and secondary metabolites (SMs) in fungi. Lysine succinylation (Ksuc) is an emerging protein PTM characterized by the addition of a succinyl group to a lysine residue, which induces substantial alteration in the chemical and structural properties of the affected protein. This chemical alteration is reversible, dynamic in nature, and evolutionarily conserved. Recent investigations of numerous proteins that undergo significant succinylation have underscored the potential significance of Ksuc in various biological processes, encompassing normal physiological functions and the development of certain pathological processes and metabolites. This review aims to elucidate the molecular mechanisms underlying Ksuc and its diverse functions in fungi. Both conventional investigation techniques and predictive tools for identifying Ksuc sites were also considered. A more profound comprehension of Ksuc and its impact on the biology of fungi have the potential to unveil new insights into post-translational modification and may pave the way for innovative approaches that can be applied across various clinical contexts in the management of mycotoxins.

## 1. Introduction

Although fungi play a crucial role in maintaining life on Earth through their involvement in nutrient recycling and their current and prospective applications in biotechnology, they also pose significant challenges across various facets of life [1]. Global health faces significant threats from fungal pathogens, impacting over 300 million individuals annually [2]. In recent times, the prevalence of invasive fungal infections has surged by more than 200%, with mortality rates for patients ranging from 30% to 90% [3]. These infections disproportionately affect immunocompromised individuals, encompassing those with HIV/AIDS, cancer patients undergoing immunotherapy, organ transplant recipients on immunosuppressive regimens, and the elderly [4]. Effectively treating fungal infections proves challenging due to a limited arsenal of antifungal drugs with low host toxicity and clinical efficacy, coupled with the emergence of drug-resistant strains [5,6,7,8]. *Candida* spp., *Aspergillus* spp. and *Cryptococcus* spp. are the most widespread opportunistic fungi responsible for global infections in immunocompromised or immunosuppressed patients [9]. These fungal infections cause over a million deaths annually, costing billions of dollars in medical expenses and imposing a significant financial burden [10].

In the last decade, interesting findings have unveiled a vast human proteome that cannot be solely explained by the genome, indicating that a single gene is capable of encoding multiple proteins that further undergo modifications to exhibit diverse functions. Two primary mechanisms are employed to achieve this proteome expansion: post-transcriptional modifications and post-translational modifications (PTMs) [11,12]. PTMs involve the covalent addition of a specific chemical group to an amino acid residue within a protein. This modification alters the chemical environment of the modified residue, which can directly or indirectly impact the protein’s functions [12]. Enzymes that add or remove such chemical groups are intricately regulated within the cells, constituting a fundamental mechanism through which cells control processes like gene transcription and protein activity [13]. Protein post-translational modification is an evolutionarily conserved mechanism in organisms, allowing them to swiftly respond to various physiological conditions, which is considered a potent biological strategy to enhance proteome diversification. PTMs encompass the covalent or noncovalent addition of functional groups to proteins, protease-mediated protein cleavage, or complete protein degradation [14]. Phosphorylation (phosphoryl group addition), acetylation (acetyl functional group introduction), ubiquitination (ubiquitin protein attachment), glycosylation (glycosyl donor addition), methylation (methyl group addition), and other events contribute to these modifications. Microbial pathogens utilize PTMs to regulate protein production and activity during infection, manipulating host proteins for disease progression [15,16,17]. Notably, fungal pathogens exploit PTMs to boost pathogenicity and facilitate fungal survival [14,18].

Presently, more than 300 types of PTMs have been discovered, occurring in both normal physiological processes and various disease conditions. Among these PTMs, methylation, acetylation, and phosphorylation have been extensively investigated [19]. Growing evidence indicates that cells meticulously regulate PTMs, leading to diverse effects on protein function and cellular structure [20,21,22]. The understanding of PTMs has undergone a significant transformation since the discovery of lysine succinylation (Ksuc). Succinylation, a conserved PTM, involves the transfer of succinyl groups (-CO-CH_2_-CH_2_-CO_2_H) from succinyl-CoA to a lysine residue on a protein. This transfer results in a mass shift of 100.0186 Da. Consequently, it converts the lysine, which originally had a +1 charge, to succinyllysine with a −1 charge, through enzymatic or non-enzymatic processes. In contrast to acetylation or methylation, which involves the addition of a 42 Da or 14 Da group, respectively, succinylation induces more significant alterations in both structure and charge, which is considered to have more substantial regulatory impacts on physiological and pathological processes [23,24]. Of all the common amino acids found in proteins, lysine is the most frequently targeted residue for succinylation. This modification leads to the conjugation of a lysine residue with the substantial molecular weight of a succinyl group, resulting in notable alterations in the protein structure [25]. This newly recognized PTM has attracted the attention of researchers due to its regulatory implications in a wide array of biological processes. Lysine succinylation now stands alongside extensively studied modifications like acetylation and methylation, contributing to the intricate web of cellular regulatory networks [20].

The exploration of lysine succinylation began with advancements in mass spectrometry-based proteomics, providing researchers with the tools to systematically examine protein modifications on a global scale. This technological progress has led to the identification of numerous succinylated proteins across diverse organisms, revealing the ubiquity and significance of this PTM [26]. Notably, in the realm of fungal biology, the complex interplay between lysine succinylation and cellular processes, including metabolism and stress response, has taken center stage [27]. The regulation of Ksuc involves a multifaceted process that includes enzymes responsible for both the addition and removal of succinyl groups. Sirtuins, a class of NAD+-dependent deacylases, assume a pivotal role in dynamically controlling succinylation levels, introducing an additional layer of complexity to the regulatory landscape [28].

Post-translational modifications (PTMs) play a crucial role in protein functionality and the control of various cellular processes and secondary metabolites (SMs) in fungi [1]. Despite numerous studies have been conducted on the production of these secondary metabolites, some of the molecular mechanisms (such as post-translational modifications, transcription factors, and various omics approaches like transcriptomics, proteomics, and metabolomics) related to the growth of the fungi and mycotoxin production remain inadequately comprehended and continue to be the focus of considerable attention [2].

To comprehend the significance of lysine succinylation (Ksuc), as a post-translational modification, in modulating the structure and functionality of diverse proteins, gaining a deeper understanding of the process becomes imperative. Thus, this review aims to emphasize the growing body of evidence that underscores the role of Ksuc in fungi, supported by biochemical, physiological, and pathological investigations. Additionally, we explore the various research approaches employed to study Ksuc.

## 2. Post-Translational Modifications (PTMs)

PTMs are essential factors that influence protein functions, regulating a wide array of physiological and pathological processes. They hold importance for protein activity, stability, folding, and localization within the cell [29]. These modifiers, originating from metabolic processes, frequently establish connections between metabolism and the modified functionality of proteins and pathways. Acetylation, methylation, biotinylation, ubiquitination, butyrylation, propionylation, crotonylation, glutarylation, malonylation, long-chain fatty acylation, ubiquitination, 2-hydroxyisobutyrylation, phosphorylation, and succinylation are among the common types of PTMs. Efforts to comprehensively identify the targets of these PTMs and understand their functional implications are still in their infancy. These modifications encompass the covalent addition of acetyl, palmitoyl, succinyl, or small ubiquitin-like modifier (SUMO) groups [29].

**Figure 1:**
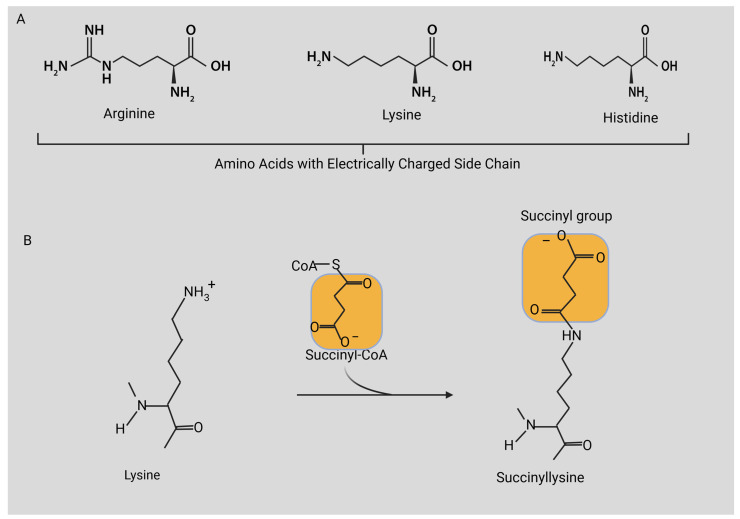
(**A**) Amino acids with electrically charged side chains. (**B**) Alteration of sizes and charges of proteins by succinylation. This modification increases the protein’s mass by 100 Da and shifts its charge from a positive to a negative state. Figure created with BioRender.com.

Among the 20 common amino acid residues utilized by ribosomes, lysine undergoes the most frequent post-translational modifications, which have significant functional and regulatory implications. At physiological pH, lysine residues possess positively charged side chains, distinguishing them as targets for a variety of PTMs. Notably, lysine is one of only three amino acids with charged side chains (Figure 1A). Engaging in diverse noncovalent interactions, the side chains can participate in van der Waals interactions, hydrogen bonds, and electrostatic interactions with negatively charged residues [25]. Creating leucine zipper structures is significantly influenced by the formation of salt bridges between lysine residues and acidic residues, serving as a notable example. In enzymatic reactions necessitating proton transfer, lysine residues assume crucial roles as participants in acid–base catalysis. Significant changes in protein function are well documented in a vast body of literature, resulting from the neutralization of the basic side chain charge of lysine through modifications like acetylation, ubiquitination, or methylation [25,30,31]. Succinylation, a relatively newly identified and less extensively examined PTM [25,32], stands out as distinctive because succinylation (100 Da) triggers a more substantial mass alteration in comparison to methylation (14 Da) or acetylation (40 Da). This modification changes a positively charged side chain to a negatively charged one, leading to a two-unit shift in the charge of the modified residues (Figure 1B).

PTMs regulate various cellular processes, influencing how fungi respond to stress and adapt to host environments. One pivotal aspect of this adaptation is the fungal cell wall, which acts as the first line of interaction between the pathogen and the host. The cell wall plays a crucial role in mediating stress responses, serving as a dynamic interface that directly influences the pathogen’s ability to survive and thrive within the host environment. The cell wall plays a protective role by triggering various pathways in response to stressors like osmotic stress, changes in pH, temperature variations, or stress induced by drugs [27,33]. In reaction to such stresses, the fungus exhibits responses such as biofilm formation, enlargement of the capsule, filamentation, and production of melanin—essential virulence factors for fungi. The cell wall’s integrity is preserved through multiple kinase-dependent pathways, including the cell wall integrity (CWI) pathway, responsible for controlling cell wall biosynthesis and repair; the high osmotic glycerol (HOG) pathway, which governs the cellular response to osmotic stress; and the mitogen-activated protein kinase (MAPK) signaling pathway, crucial for environmental adaptation [27,34,35,36,37].

On the other hand, the calcineurin signaling pathway, which is a protein phosphatase pathway regulated by intracellular Ca^2+^, is involved in stress response [38]. The activation of pathways associated with the cell wall through phosphorylation is instrumental in conferring fungal tolerance to the host environment. A comprehensive examination of the global phosphoproteome in *Aspergillus fumigatus* showcased the fungal reaction to cell wall stress induced by Congo red, a cell wall-damaging agent. This analysis identified 485 proteins that are potentially implicated in the response to cell wall damage [39]. This investigation centered on the function of two MAPKs, SakA and MpkC, which act as key regulators in the HOG pathway and play essential roles in the tolerance to antifungal agents (such as caspofungin). The proteins phosphorylated during cell wall stress encompassed those involved in signal transduction, stress response, protein kinases, actin cytoskeleton, budding cell polarity, and filamentation. This research work underscored the participation of the HOG pathway in responding to cell wall stress and identified several novel proteins crucial for preserving the cell wall. This suggests potential new components in fungal signaling processes that could be targeted by novel antifungal agents. The authors’ subsequent work aims to characterize osmotic and cell wall stress kinases and transcriptional factors, presenting an opportunity for intervention in critical fungal signaling events.

The stress response in fungi is additionally governed by the activities of acetyltransferases, which facilitate the PTM of acetylation. A recent discovery unveiled a connection between the histone acetyltransferase (HAT) GcnE and vital developmental and regulatory processes in *A. fumigatus*, including growth, biofilm formation, and stress tolerance [40]. The acetyltransferase Gcn5 (known as GcnE in *A. fumigatus*) belongs to the evolutionarily conserved Gcn5-related N-acetyltransferase family (GNATs) and is a component of the large transcriptional multiprotein complex Spt-Ada-Gcn5 acetyltransferase (SAGA) [41].

Accumulating evidence strongly indicates the indispensability of post-translational modifications (PTMs) in the growth and developmental processes of fungi, particularly in the regulation of crucial cellular mechanisms contributing to fungal pathogenicity. One validated example is the role of N-glycosylation in fungal pathogenesis. The α-1,6-mannosyltransferase Och1 plays a pivotal role in initiating the formation of a distinct branch on the N-glycan core, facilitating the subsequent addition of mannosylated outer chains. *Candida albicans* mutants deficient in Och1 exhibit significant cell wall defects and reduced virulence [42]. Another instance involves protein O-mannosyltransferases, which play a key role in initiating the biosynthesis of O-mannosyl glycans in the endoplasmic reticulum (ER). In *S. cerevisiae*, triple mutants of PMTs result in lethality [43], and the absence of PMTs causes diminished virulence in both *C. albicans* and *C. neoformans* [44,45,46,47]. Furthermore, the regulation of ubiquitination in fungal pathogens plays a role in stress adaptation, metabolism, morphogenesis, and various developmental processes [14].

Xu et al. identified 24 succinylated proteins associated with pathogenicity in *Trichophyton rubrum* or homologous proteins participating in virulence in other fungi [48]. Proteases that are secreted and play a crucial role in digesting hard keratin tissues during infection are essential for the virulence of dermatophytes. Among the succinylated proteins, eight are secreted proteases, including aminopeptidase, aspartic endopeptidase Pep2, leucine aminopeptidase 1, leucine aminopeptidase 2, subtilisin-like protease, Peptidase S41 family protein, tripeptidyl-peptidase SED2, and carboxypeptidase S1. In addition to secreted proteases, the mdr2-encoded ABC multidrug transporter (succinylated at K361 and K368) and AcuE-encoded malate synthase (succinylated at K161, K319, K483, K486, and K501) are also implicated in dermatophyte infection. In their study, two Rho-type GTPases associated with fundamental growth processes, namely Rho GTPase Rho1 and Rho-GDP dissociation inhibitor (Rho-GDI), were identified as being succinylated [49,50,51]. Rho-type GTPases play a regulatory role in various essential growth processes, including cytoskeletal arrangement, vesicle trafficking, cell wall biosynthesis, and polarized growth. Additionally, they have been implicated in fungal infection [52,53,54]. Moreover, there is an association between heat shock proteins (Hsps) and fungal pathogenicity. Beyond their function as chaperone proteins, Hsps play specific roles in fungal processes like dimorphic transition, drug resistance, and virulence. For instance, Hsp90, which is implicated in morphogenesis, antifungal resistance, and fungal pathogenicity, is considered a potential target for antifungal therapy [55]. The four Hsps identified in their study, namely Hsp31, Hsp60, Hsp70, and Hsp90, exhibited substantial succinylation. These pivotal proteins crucial for pathogenicity have been identified as succinylation targets.

Similarly, significantly elevated succinylation levels were observed in *A. flavus* when cultured in an enhanced medium comprising sodium succinate, sodium acetate, sucrose, and sodium chloride, in comparison to *Aspergillus flavus* grown in a yeast extract-supplemented medium. This indicates that distinct growth media could potentially modify the lysine succinylation profile [56]. In the analysis of function, the Ksuc levels of the immunoprecipitated versicolorin B synthase (VBS) from wild-type [57] VBS and its mutants (K135A and K135R) were compared. It was subsequently validated that the activity of VBS is notably dependent on K135, emphasizing the significance of succinylation at K135 in the maintenance of sclerotia and AF production [58]. In summary, PTMs have vital functions in priming fungal cells for high-stress conditions, particularly during host infection. This involves the control of signaling cascades, protein degradation, and protein activation.

## 3. Protein Lysine Acylation

Protein lysine acylation is widely established as a vital regulator of various protein functions, which include modulating chromatin structure, controlling gene expression, influencing enzyme activity, and affecting protein–protein interactions. Due to its positively charged side chain, lysine residues are potent targets for various PTMs, such as the attachment of acetyl, succinyl, or methyl groups. These alterations, which impact the distribution of electrical charge on the lysine residue, can exert substantial structural and functional influences on the associated protein [59]. Presently, in mammals, researchers have identified over ten distinct forms of lysine acylation, which include malonylation [32], succinylation [25], glutarylation [60], crotonylation [61], propionylation, butyrylation [62], 2-hydroxyisobutyrylation [63,64] and lactylation [65], among others. Additionally, a multitude of proteins in microorganisms like those in the gut microbiota exhibit significant acylation [66]. Notably, the anionic acylations, including malonylation, succinylation, and glutarylation, have garnered significant interest as crucial post-translational modifications conserved from bacterial to mammalian species [25,67,68]. Notably, acetylation of lysine residues, a widely studied phenomenon, has been shown to influence various intricate processes, including enzymatic activity, transcriptional regulation, and protein degradation [69]. The study of acetylation has prompted further investigations into other acyl modifications of lysine. Recent investigations have brought to light additional modifications of the lysine side chain, including malonylation, succinylation, and glutarylation [25,32]. These modifications are evolutionarily conserved and can be found in various cellular pathways, with a notable enrichment in metabolic pathways [60,68]. Unlike acetylation, these three acidic PTMs add a negative charge to the positively charged ε-amino group of lysine. While their addition appears to be regulated through nonenzymatic means, specific KDACs from the Sirtuin family catalyze their removal.

Malonylation of lysine was initially detected on non-histone proteins [32], and it was subsequently found on histones as well [67]. This groundbreaking research provided evidence that Sirt5, belonging to the class III lysine deacetylases, can act as a demalonylase both within laboratory settings and a living organism [32]. Proteomic analysis demonstrated that the majority of substrates for lysine malonylation are metabolic enzymes. Biochemical investigations have substantiated the involvement of lysine malonylation in various metabolic pathways, including mitochondrial respiration [70], fatty acid metabolism [71], and glycolytic pathways [72]. However, much research is needed to elucidate this protein lysine acylation in fungi.

**Figure 2:**
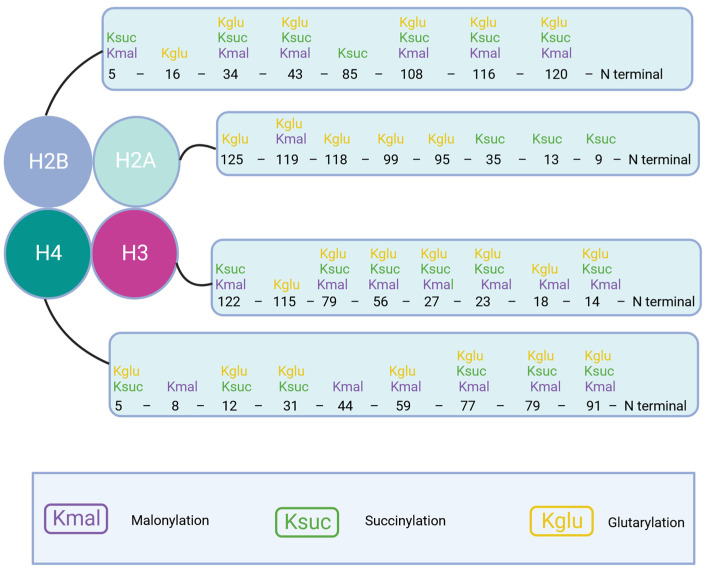
Distribution of different forms of protein lysine acylation (lysine malonylation, lysine succinylation, and lysine glutarylation) on histones in the nucleosome. Figure created with BioRender.com.

In terms of their chemical makeup, lysine succinylation and lysine malonylation are closely related PTMs, differing by just one carbon in the acyl chain. The enzyme pocket of Sirt5 is capable of recognizing both succinylated lysine and malonylated lysine, and it can catalyze the removal of these acylations from lysine residues [73]. However, when examined through proteomic profiling, it becomes evident that the lysine malonylome [72] differs significantly from the lysine succinylome [74]. This observation implies that lysine succinylation and malonylation likely possess distinct functions and regulatory mechanisms (Figure 2).

In 2014, the identification of lysine glutarylation [60] broadened our understanding of acidic lysine acylations, adding to the diversity of this type of modification. Glutaryl-CoA, a vital intermediate in amino acid metabolism, serves as a cofactor in this modification process. A comprehensive examination of the proteome revealed that this epigenetic modification is abundant in metabolic enzymes and mitochondrial proteins. This highlights the substantial functional roles of lysine glutarylation in cellular regulation and diseases [23,71]. In comparison to histone lysine malonylation and succinylation, it is believed that histone lysine glutarylation exerts the most significant influence on nucleosome structure and dynamics due to its longer acyl chain (Figure 2).

The reactions involving lysine malonylation, succinylation, and glutarylation all rely on the respective acyl-CoA as the cofactor that donates the acyl group. These acyl-CoAs play significant roles as intermediates in cellular metabolism. For instance, malonyl-CoA can be synthesized from glucose through de novo pathways [75]. Succinyl-CoA serves as a crucial intermediate in the tricarboxylic acid (TCA) cycle [76]. Additionally, glutaryl-CoA is implicated in amino acid metabolism. Hence, these acyl-CoAs act as intermediaries linking cellular metabolism and the epigenome [77]. Research findings have supported the direct relationships between cellular concentrations of specific acyl-CoAs and the prevalence of related histone acylations [78]. Research has shown that histones can function as detectors of cellular acetyl-CoA levels, modulating chromatin structure and gene expression [79,80,81]. However, the precise lysine acylation metabolic control of chromatin dynamics and their functions in physiological processes and disease development remain elusive and have not been identified in fungi. The following section provides detailed information about the discovery of lysine succinylation.

## 4. Discovery of Lysine Succinylation

The identification of lysine succinylation originated from in-depth investigations into acetylation. The initial utilization of succinylation in 1961 involved employing it as a technique to hinder the production of antibodies and subsequently assess erythematous reactions in animals susceptible to dinitrophenyl-polyline [82]. A study conducted in 1975 examined the impacts of lysine succinylation on ovalbumin protein, and the findings revealed that succinylation induced by succinic anhydride led to a change in the protein’s conformation. They also suggested a potential link between succinylation and the protein’s resistance to digestion by tryptic attack [83]. In 2004, a study further explored the interaction between succinyl groups and the amino acid homoserine using homoserine trans-succinylase [84]. This study revealed the essential role of lysines on the enzyme for succinyl binding, which motivated the further impact of lysine succinylation on various proteins [84]. In 2011, through the application of affinity purification using an anti-succinyl lysine antibody, the natural occurrence of succinylation on lysine residues in bacteria was unveiled [25]. For the preliminary detection of the succinyllysine residue, they employed mass spectrometry and the alignment of protein sequences. Mass spectrometry combined Western blot analysis, in vivo labeling with isotopic succinate, and HPLC-MS/MS to authenticate the presence of succinyllysine peptides derived from proteins within the organism. Moreover, succinyl coenzyme A [26], a crucial metabolic intermediate, is believed to potentially act as a cofactor for Ksuc, implying that Ksuc could potentially impact various cellular metabolic pathways. Additionally, under physiological pH conditions (7.4), Ksuc alters the charge of lysine from +1 to −1, leading to a significant modification in the protein’s structure. Given the substantial prevalence of Ksuc and the resulting biochemical alterations it triggers; this modification has garnered growing interest among researchers. In the subsequent years, multiple investigations identified proteins related to succinylation, including histones, the specific sites on proteins, and several succinylases contributing to its regulation [11,26,28,67,68,71,73,74,85]. Remarkably, these recent findings suggest that succinylation could have significance in conditions related to the disruption of mitochondrial function, like those linked to oxidative stress [11,86,87,88]. In 2018, a global succinylome analysis in *Aspergillus flavus* was conducted. This involved utilizing high-precision nano-LC-MS/MS, along with the isolation of succinylated peptides from lysed cells and their subsequent identification [56]. The following section provides detailed information about the biochemical process of succinylation modification.

## 5. Biochemical Process of Succinylation Modification

### 5.1. Non-Enzymatic Lysine Succinylation by Succinyl-CoA

The succinyl group in Ksuc can originate directly from succinyl-CoA through a nonenzymatic process, as demonstrated in prior research [89,90]. Dependence on succinyl-CoA from the TCA cycle for succinylation in yeast cells was demonstrated by Weinert et al. Their analysis spanned various organisms and utilized succinyl-CoA as the lysine succinyl donor at concentrations influencing succinylation levels [68]. Primarily originating within the mitochondria via odd-numbered fatty acid oxidation or the TCA cycle, the synthesis of succinyl-CoA is prominent. A previous investigation highlighted that succinic acid can cross the mitochondrial membrane, influencing cytoplasmic Ksuc levels [91]. Intriguingly, within the cytoplasm, investigators have also identified succinyl-CoA synthetase dependent on GTP [92]. This suggests that succinyl-CoA could be present in various cellular compartments including both within and outside mitochondria, implying that the process of Ksuc regulated by succinyl-CoA takes place across different cellular regions. Later investigations indicated that over one-third of nucleosomes, encompassing both histone and non-histone chromatin components, displayed succinylation. This finding underscores the potential influence of chromatin succinylation on nuclear gene expression [93]. In a separate study, it was demonstrated that a mere 8% of yeast’s succinylated proteins were produced within the mitochondria. Interestingly, the predominant occurrence of lysine succinylation was observed in the cytosol and nucleus, primarily facilitated by succinic acid or related metabolites. The prevalence of lysine succinylation beyond the confines of mitochondria suggests the potential influence of succinyl-CoA, succinic acid, or related metabolites on cytosolic and nuclear succinylation. This insight emerged from the consistent presence of Ksuc outside mitochondrial boundaries [68]. Likewise, the work of Zhang et al. showed how succinic acid, derived from the tricarboxylic acid cycle, can act as a succinyl group reservoir through isotope labeling techniques [25]. In addition, succinylation within yeast or *Escherichia coli* cells is induced by succinyl-CoA, which is derived from the TCA cycle. The concentration of succinyl-CoA plays a significant role in regulating the overall levels of succinylation. Furthermore, Ren et al. revealed that almost all of the enzymes participating in the TCA cycle of *Aspergillus flavus* were identified as targets of lysine succinylation. These findings also imply a possible role for lysine succinylation in regulating the activity of these enzymes within the TCA cycle [56]. In summary, these investigations collectively suggest that Ksuc might be influenced by succinyl-CoA, succinic acid, or other succinyl-metabolites, exerting their impact both within and beyond the confines of mitochondrial environments.

### 5.2. Enzymatic Lysine Succinylation

**Figure 3:**
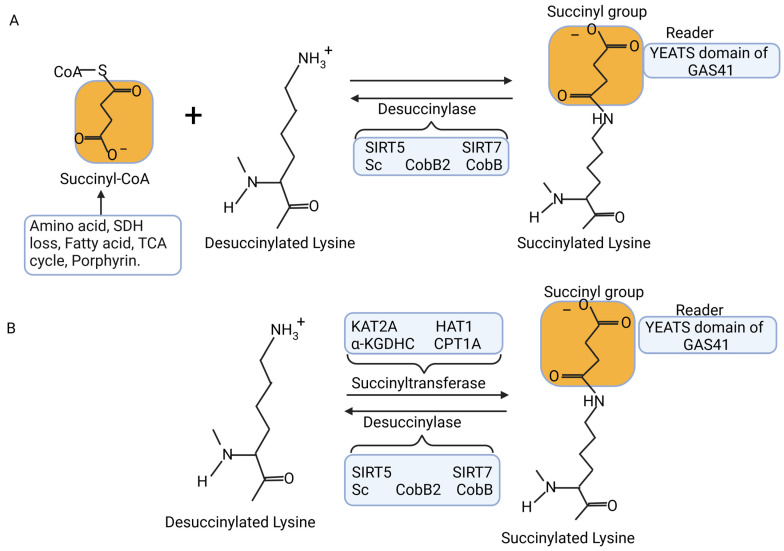
Process of lysine succinylation, which involves the covalent attachment of succinyl groups (-CO-CH_2_-CH_2_-CO_2_H) to protein lysine residues. This process can occur through enzymatic or non-enzymatic mechanisms. (**A**) Non-enzymatic reaction: Succinyl donor succinyl-CoA plays a role in modifying lysine residues of proteins through succinylation. Succinyl-CoA primarily originates from various metabolic pathways, including the tricarboxylic acid (TCA) cycle, fatty acid metabolism, amino acid metabolism, and porphyrin metabolism. The levels of succinylation modification are also regulated through desuccinylation by enzymes like desuccinylase, CobB, ScCobB2, as well as SIRT5 and SIRT7. (**B**) Enzymatic reaction: Some succinyltransferases, such as KAT2A, HAT1, a-KGDHC, and CPT1A, are capable of catalyzing lysine succinylation within cells. These enzymes contribute to the succinylation process. Additional proteins and domains involved in this process include succinate dehydrogenase (SDH), tricarboxylic acid (TCA), sirtuin 5 (SIRT5), sirtuin 7 (SIRT7), α-ketoglutarate dehydrogenase complex (α-KGDHC), lysine acetyltransferase 2A (KAT2A), histone acetyltransferase 1 (HAT1), carnitine palmitoyltransferase 1A (CPT1A), glioma amplified sequence 41 (GAS41), and the YEATS (Yaf9, ENL, AF9, Taf14, Sas5) domain. Figure created with BioRender.com.

In addition to the succinylation linked to succinyl-CoA and succinyl metabolites through non-enzymatic processes, certain scientists have detected and characterized enzyme-driven succinylation within cellular environments (Figure 3). Gibson and colleagues discovered that the alpha-ketoglutarate dehydrogenase complex (α-KGDHC) functions as a transsuccinylase, facilitating the succinylation of several mitochondrial proteins, including those involved in the TCA cycle. This modification alters the function of these proteins in an alpha-ketoglutarate-dependent manner. Notably, the efficiency of α -KGDHC relies on the E2k subunit, which exhibits greater activity in comparison to succinyl-CoA [94]. Wang and colleagues documented that the alpha-ketoglutarate dehydrogenase complex (α-KGDHC) can associate with lysine acetyltransferase 2A (KAT2A), an enzyme responsible for histone succinylation. Subsequently, KAT2A can succinylate histone H3 at lysine 79 (H3K79). This modification predominantly takes place in proximity to the transcriptional initiation site of genes [95]. Importantly, it was observed that KAT2A plays a role in regulating the succinylation of H3K79 in the promoter region of the YWHAZ gene, responsible for encoding the 14-3-3ζ protein. This regulation leads to an increase in the expression of both 14-3-3ζ and YWHAZ mRNA, subsequently preventing the degradation of beta-catenin in pancreatic ductal adenocarcinoma (PDAC) cells [96]. This indicates a potential association between protein succinylation and the development or progression of cancer. Wang et al. discovered that the YEATS (Yaf9, ENL, AF9, Taf14, Sas5) domain of GAS41 (glioma amplified sequence 41) exhibits strong binding to H3K122 when a histidine residue is protonated. They also observed the simultaneous presence of GAS41 and H3K122suc within the pH range of 6.0 to 7.0. As the pH value rose to 7.4, the interaction between GAS41 and H3K122suc notably diminished, while there was a concurrent rise in H3K122cr (crotonylation) and H3K122ac (acetylation). This suggests that the GAS41 YEATS domain functions as a pH-dependent reader of histone Ksuc modifications [97]. Furthermore, Kurmi et al. identified lysine succinyltransferase (LSTase) activity in carnitine palmitoyltransferase 1A (CPT1A), both in vivo and in vitro. They noted that in WT-CPT1A cells, 171 lysine sites on 101 proteins (out of 550 total lysine sites on 247 proteins) exhibited hypersuccinylation. Enolase 1 stood out as one of the highly succinylated proteins, and this modification led to a decrease in enolase enzymatic activity within the cells. Later on, they discovered that introducing a mutation (G710E) in CPT1A specifically deactivated carnitine palmitoyltransferase (CPTase) function while leaving LSTase activity unaffected. This mutation (G710E) led to a reduction in enolase activity and enhanced cell proliferation when glutamine was scarce, all without affecting cellular succinyl-CoA levels. These findings imply that CPT1A, acting as an LSTase, controls the enzymatic activity of proteins and cellular metabolism independently of its CPTase function [98]. CPT1A has also been observed to control the succinylation of S100A10 at lysine residue 47 (K47) within gastric cancer cells. The succinylation of K47 in S100A10 resulted in the enhancement of the protein’s stability by preventing its ubiquitination and subsequent proteasomal degradation via the proteasome [99]. Furthermore, the synthesis of aflatoxin (AF) is inversely associated with the extent of succinylation observed in proteins related to metabolism in the high-aflatoxin strain/low-aflatoxin strain (H-AFs/L-AFs) [58]. Intriguingly, Ren et al. observed that *Aspergillus flavus*, when exposed to sodium succinate, displayed increased succinylation of total proteins but experienced a substantial reduction in aflatoxin production [56]. This indirectly indicates that the overall suppression of succinylation influences AF synthesis. The biosynthesis of AF requires the involvement of a minimum of 17 enzymes that result from the expression of approximately 25 or more genes concentrated within a 75 kb section on a single chromosome [100]. Aflatoxins are furanocoumarins derived from polyketides and are initially generated through the conversion of acetyl-CoA to malonyl-CoA, a process catalyzed by acetyl-CoA carboxylase. Subsequently, Fas-1 and Fas-2-driven catalysis is responsible for creating the initial hexanoate unit [101]. Wang et al. [58] notably observed the downregulation of succinylation in acetyl-CoA carboxylase, Fas-1, and Fas-2 in H-AFs. These findings imply that Ksuc plays a significant role as a post-translational modification in the secondary metabolic pathway of AF synthesis. It potentially exerts direct control over the structure or functionality of the enzymes responsible for the initial phase of AF synthesis [58].

Wang et al. [58] performed functional analysis in which they compared the Ksuc levels between immunoprecipitated VBS and wild-type VBS and its mutants (K135A and K135R). This comparison provided further confirmation that K135 plays a crucial role in VBS activity. Moreover, succinylation of K135 emerged as a pivotal factor in sustaining the formation of sclerotia and AF production [58].

### 5.3. Enzymatic Desuccinylation

As the investigation into succinylation modification progressed, researchers uncovered several desuccinylases that play a role in its negative regulation. In 2013, researchers led by Colak identified Cob B, the first bacterial lysine deacetylase with sirtuin2-like properties, as a prokaryotic desuccinylase possessing both deacetylation and desuccinylation activities [28]. Likewise, a sirtuin-like protein called ScCobB2 was identified as a distinct desuccinylase in Streptomyces coelicolor [102]. In the same vein, desuccinylases in eukaryotes, namely sirtuin 5 (SIRT5) [74] and sirtuin 7 (SIRT7) [103], were discovered (Figure 3).

Numerous investigations have demonstrated that SIRT5 functions as a lysine deacylase dependent on NAD^+^, facilitating the selective desuccinylation of different histone succinyl sites. This enzyme plays a significant role in various physiological processes within the cell. Notably, the recognition of SIRT5 and succinyl peptides relies on crucial conserved main chain hydrogen bonds formed by the succinyl lysine (0), +1, and +3 sites, as demonstrated by various studies [73,104]. Four distinct isoforms of the SIRT5 protein, denoted as SIRT5^iso1–4^ are encoded by the SIRT5 gene. In humans, the SIRT5 gene produces four protein isoforms known as SIRT5^iso1–4^. While SIRT5^iso1–3^ is situated in the mitochondria, SIRT5^iso4^ predominantly resides in the cytoplasm. Notably, among these isoforms, SIRT5^iso1^ exhibits greater deacylase activity compared to SIRT5^iso2–4^ [73].

Cellular processes like fatty acid metabolism and amino acid metabolism are significantly impacted by the essential involvement of SIRT5. Investigations involving the overexpression of hepatic SIRT5 in ob/ob mice revealed noteworthy findings. These mice exhibited lowered levels of succinylation and malonylation, heightened fatty acid oxidation, and an ameliorated hepatic steatosis state in comparison to healthy mice. This outcome suggests that the enhancement of SIRT5 through hepatic overexpression leads to the mitigation of metabolic disorders in these mice using protein desuccinylation [105]. The promotion of fatty acid oxidation is facilitated by SIRT5-mediated desuccinylation, which heightens the activity of the α subunit of enoyl-CoA hydratase (ECHA) at K351 [106]. Furthermore, SIRT5 can suppress the function of Acyl-CoA oxidase 1 (ACOX1), leading to an impact on fatty acid oxidation processes. This indicates that SIRT5 extends to the modulation of ACOX1’s activity and involvement in fatty acid oxidation [107]. It is intriguing to note that when UCP1 (uncoupling protein 1) experiences heightened succinylation due to SIRT5 regulation within brown adipose tissue, its function and stability substantially decline. This alteration results in compromised mitophagy, disrupted mitochondrial respiration, and perturbed lipid metabolism, showcasing the intricate impact of succinylation on these processes [108].

Glycometabolism is another domain where SIRT5’s involvement is evident. Within this context, it exerts its influence by dampening the activity of pyruvate kinase M2 (PKM2). This reduction is achieved by diminishing the level of succinylation at K498, resulting in the inhibition of pyruvate production through PKM2 [108]. By curtailing the succinyl binding to the E1α subunit, SIRT5 possesses the ability to modulate the oxidative breakdown of glucose, consequently leading to the suppression of pyruvate dehydrogenase complex (PDC) activity. This underlines SIRT5’s regulatory role in aerobic glucose oxidation [109]. Furthermore, the desuccinylation action of SIRT5 extends to K547 within the subunit A of the succinate dehydrogenase complex (SDHA), a key player in the tricarboxylic acid (TCA) cycle. Remarkably, the suppression of SIRT5 leads to the revival of SDHA’s activity and the subsequent surge in succinylation levels, amplifying the significance of SIRT5’s role [110]. Notably, SIRT5 assumes significant roles in amino acid metabolism as well. It enhances serine catabolism by desuccinylating serine hydroxymethyltransferase (SHMT2) at K280, which in turn upregulates its activity [111]. Conversely, SIRT5 suppresses the synthesis of glutamine through the process of desuccinylating glutaminase (GLS) [112]. In the oxidative phosphorylation pathway, succinylation stands as the preferred target for SIRT5 [105]. Through its interaction with cardiolipin, SIRT5 enhances the function of the respiratory chain by acting on protein complexes situated on the inner mitochondrial membrane [113]. When SIRT5 is deficient, it results in the suppression of ATP synthase activity and inhibition of mitochondrial NADH oxidation, ultimately impacting ATP production within the mitochondria [114]. Furthermore, the regulation of 3-hydroxy-3-methylglutaryl-CoA synthase 2 (HMGCS2), a pivotal enzyme in ketogenesis, is modulated by SIRT5 through its control of succinylation. This influence on HMGCS2 highlights SIRT5’s role as a key regulator of ketogenesis, both in vivo and in vitro [74]. Additionally, SIRT5 possesses the ability to catalyze the desuccinylation and subsequent to activate isocitrate dehydrogenase 2 (IDH2) and Cu/Zn superoxide dismutase (SOD1), consequently resulting in reduction in reactive oxygen species (ROS) levels and reinforcing the cellular antioxidant defense mechanisms [115,116].

Although SIRT7 has been identified as a NAD-dependent histone deacetylase, the precise enzymatic activity and cellular role of SIRT7 are still not fully understood. Li and colleagues uncovered that SIRT7 is drawn to DNA double-strand breaks (DSBs) through dependence on poly (ADP-ribose) polymerase 1 (PARP1). In this context, it conducts the desuccinylation (the removal of succinyl groups) of H3K122, which then fosters processes like DSB repair and chromatin condensation [103].

Furthermore, enzyme desuccinylation was conducted in *Aspergillus flavus*. The findings indicated that altering the K370 residue to either arginine or alanine, mimicking the state of desuccinylation in AflE, significantly impacted aflatoxin production. This suggests that K370 succinylation can affect aflatoxin biosynthesis by modifying the function of AflE in *Aspergillus flavus* [56]. Given the notable alterations in sclerotial development observed in the absence of *aflE* or when K370 was replaced with R or A to mimic desuccinylation, they concluded that K370 desuccinylation is necessary for the proper regulation of AflE in the sclerotial development of *Aspergillus flavus* [56]. In another study, the desuccinylation mimic (ΔvbsK135A/R) displays notable differences compared to the normal strain, suggesting that succinylation plays a role in influencing the production and development of *Aspergillus flavus* sclerotia [58].

The regulation of succinylation involves two enzyme types, lysine succinyltransferases and desuccinylases. Currently, there are no reported enzymes in fungi that perform both succinylation and desuccinylation. The only enzyme identified to date with lysine succinyltransferase activity is the histone acetyltransferase p300 [23]. Due to the structural resemblance of these short-chain acyl-CoAs (such as malonyl-CoA, succinyl-CoA, and glutaryl-CoA) to acetyl-CoA, there was a suggestion that acetyltransferases catalyze promiscuous acyltransferase activity [23]. Sirtuins, alternatively termed Sir2 (Silent information regulator 2) proteins, constitute a category of NAD+-dependent deacetylases. Within mammalian cells, seven sirtuins (SIRT1–7) have been recognized. Existing evidence indicates that SIRT3–5 and SIRT7 possess desuccinylase activity [23] (Table 1). The following section provides detailed information about the mitochondrial and cytosol localization of lysine succinylation.

## 6. Mitochondria and Cytosol Localization of Lysine Succinylation

Within mitochondria, protein lysine succinylation is more widespread compared to its occurrence outside, and this can be attributed to the heightened generation of succinyl-CoA in the citric acid cycle. Additionally, the matrix possesses a significantly higher abundance of coenzyme A (CoASH) and acyl-CoAs compared to the cytosol [120,121,122]. Although the initial credit for succinyl-CoA production was given to the reverse action of Succinate-CoA ligase, it is now established that this phenomenon is primarily due to the activity of KGDHC during glutamate catabolism. This revelation was recently substantiated within the context of erythropoiesis [123]. This is expected to hold in various other pathological circumstances, as demonstrated by numerous studies [124,125,126]. Certainly, protein hyper-succinylation results from Succinate-CoA ligase deficiency [127]. Nevertheless, it is well established that lysine succinylation is not limited to the mitochondria and also occurs outside of it [26,68,90,127,128]. In addition, there exists lysine succinyltransferase activity associated with carnitine palmitoyltransferase (CPT), an enzyme located on the outer mitochondrial membrane facing the cytosol [99]. Yet, considering the inability of succinyl-CoA to freely pass through membranes and the lack of any established transfer mechanism [79,129], the inquiry emerges regarding how this metabolite is transported to the cytosol and the pathways involved.

**Figure 4:**
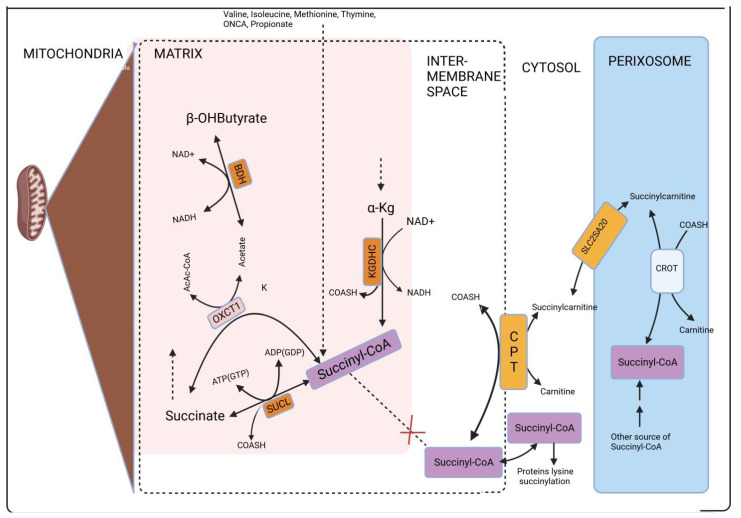
Metabolic pathways for the formation of succinyl-CoA, highlighting its production in the mitochondria, cytosol, and peroxisomes. α-Kg: alpha-ketoglutarate, BDH: beta-hydroxybutyrate dehydrogenase, β-OHbutyrate: beta-hydroxybutyrate, CoASH: coenzyme A, CPT: carnitine palmitoyltransferase (I), CROT: carnitine O-octanoyl transferase, KGDHC: alpha-ketoglutarate dehydrogenase, ONCA: odd-number chain fatty acid, OXCT1: 3-oxoacid CoA-transferase 1, SUCL: Succinate-CoA ligase, SLC25A20: mitochondrial carnitine/acyl-carnitine carrier protein. Adapted and modified with permission from [140]. Copyright © 2022, Chinopoulos. Figure created with BioRender.com.

Succinyl-CoA has the potential to appear within the cytosol, including the nucleus, via one of the following three pathways:

  i. The conversion of succinylcarnitine to carnitine and succinyl-CoA is facilitated by carnitine palmitoyltransferase [130]. This enzyme is present in three isoforms, each with a tissue-specific distribution: isoform A is found in the liver, isoform B is located in the muscle, and isoform C is situated in the brain. All three isoforms are situated in the outer mitochondrial membrane [131,132], with isoform C also being present in the endoplasmic reticulum [133]. CoA can be found in the cytosol through various pathways, whereas succinyl carnitine can be transported from peroxisomes via the carrier protein SLC25A20. Initially identified as a “mitochondrial carnitine/acylcarnitine carrier protein”, it has since been recognized to also exist in peroxisomes. In peroxisomes, succinylcarnitine synthesis occurs through the following process: carnitine and succinyl-CoA are converted into succinylcarnitine and CoA with the involvement of carnitine O-octanoyltransferase (CROT), as discussed in more detail in [57]. In the context of human peroxisomes, the necessary carnitine might be brought in through one of the organic cation transporters (OCTs). In rodents, the OCTN3 (organic cation transporter 3) carnitine transporter (SLC22A21) is present [134,135]. Nevertheless, there is some debate regarding whether OCTN3 is consistently localized in peroxisomes [136].
 ii. The transportation of succinyl-CoA from the mitochondria to the cytosol relies on a model assumption presented in [137]. This assumption was part of an effort to create a comprehensive and high-quality genome-scale metabolic reconstruction by Thiele and Palsson. However, it has not been experimentally confirmed.
iii. Adenosine triphosphate (ATP) + Coenzyme A (CoA) + succinate combine to form Adenosine monophosphate (AMP) + Inorganic pyrophosphate (PPi) + succinyl-CoA. This transformation is potentially facilitated by a microsomal dicarboxylyl-CoA synthetase (E.C. 6.2.1.23). In the rat liver and kidney, but not in the muscle tissue, dicarboxylic acids (succinate) can be converted into their CoA esters by this enzyme [138,139]. Nevertheless, there is a suggestion that this enzyme might not be effective on short-chain dicarboxylic acids like succinate. This is because enzyme activity tends to approach zero as the carbon chain length of the dicarboxylic acid decreases, and it is already minimal with a chain length of C = 5 [138] (Figure 4).

To sum up, the authors believe that the sole feasible way for succinyl-CoA to reach the cytosol is by being produced within peroxisomes and then exported in the form of succinyl carnitine. This leads to the question: how is succinyl-CoA generated within peroxisomes? Additional information regarding the generation of succinyl-CoA and the steps involved are provided in the literature [140], and interested readers are directed to these specific studies for more details.

Ren et al. employed the YLoc website to assess the subcellular localization of succinylated proteins in *Aspergillus flavus*. This analysis predicted that 146 proteins resided in the cytoplasm, 85 in the mitochondria, 71 in the nucleus, and 47 in the secretory pathway. These findings affirm the association between succinylation modification and protein function within the metabolic network [56]. In another study, around 36% (131 in total), 25% (90 in total), and 24% (85 in total) of the succinylated proteins that exhibited downregulation were situated in the cytosol, nucleus, and mitochondria, respectively, in *Aspergillus flavus* [58].

## 7. Lysine Succinylation in Fungi

Succinylation has been observed in a wide range of proteins, including both histones and non-histone proteins, and it plays a role in the regulation of diverse cellular processes [67,68]. Recently, a comprehensive investigation of the lysine succinylome was conducted in *Aspergillus flavus*. This study revealed the presence of 349 succinylated proteins within this fungus. These proteins were found to participate in various biological processes, with a particular emphasis on processes related to aflatoxin (AF) biosynthesis [56]. Within the enzymatic cascade involved in AF synthesis, the proteins AflE (AFLA_139310), AflK (AFLA_139190), and AflM (AFLA_139300) were identified as having succinylated lysine residues. The significance of lysine succinylation at the K370 site of norsolorinic acid reductase NorA (AflE) was investigated, revealing its importance for AflE activity. Additionally, 24 other lysine succinylated proteins, referred to as Ksu proteins, were found to be distributed across 20 different biosynthetic gene clusters (BGCs), including the cyclopiazonic acid cluster [56]. Given that aflatoxin biosynthesis and export processes are reliant on vesicles and endosomes, it is conceivable that lysine succinylation of the v-SNARE protein, which is associated with vesicle transport, could potentially impact aflatoxin production. Further investigation, involving site-specific mutations, provided evidence that lysine succinylation of AflE could influence the development of sclerotia and the production of aflatoxins in *Aspergillus flavus*. In total, the analysis identified 985 succinylation sites on 349 proteins that had undergone succinylation in *Aspergillus flavus*. These proteins were involved in various biological processes which encompassed translation, the production of precursor metabolites and energy, the breakdown of monosaccharides, and the catabolism of glucose [56].

Furthermore, Wang et al. conducted a proteomic quantification of lysine succinylation (Ksuc) to investigate its potential role in regulating secondary metabolism, including aflatoxin (AF) production, in *Aspergillus flavus* under natural conditions [58]. This study utilized a quantification method that involved tandem mass tag labeling and antibody-based affinity enrichment of succinylated peptides. In total, 1240 Ksuc sites within 768 proteins were identified, with 1103 sites in 685 proteins being quantified [58]. By comparing the levels of succinylated proteins between *Aspergillus flavus* strains that produced high and low amounts of AF, bioinformatics analysis revealed that the majority of succinylated proteins involved in the AF biosynthetic pathway were downregulated. This downregulation had a direct effect on AF synthesis. Specifically, versicolorin B synthase, a crucial catalytic enzyme in the synthesis of heterochrome B during AF production, was identified as succinylated. Site-directed mutagenesis and biochemical investigations further demonstrated that succinylation of versicolorin B synthase played a significant role in regulating sclerotia development and AF biosynthesis in *Aspergillus flavus*. [58].

A growing body of evidence suggests that lysine succinylation overlaps with other PTMs, such as acetylation and malonylation, at common sites. This overlap is believed to contribute to the regulation of metabolic pathways and various cellular processes [67,68]. Interestingly, out of the 26 proteins identified in BGC synthesis pathways, 20 of them are acetylated. Notably, they also share the same lysine modification site. In the model medicinal mushroom *Ganoderma lucidum*, lysine succinylation is associated with several secondary metabolites, including pharmacologically active compounds [141]. Notably, 47 succinylated enzymes participating in the biosynthesis of triterpenoids and polysaccharides in *G. lucidum* have been identified. This implies that post-translational modifications at enzymes involved in secondary metabolite synthesis might serve as an alternative regulatory mechanism for the biosynthesis of secondary metabolites in response to various environmental stimuli in fungi.

On the other hand, several investigations have been documented, unveiling the potential functions of lysine succinylation (Ksuc) in various fungi, encompassing *Pyricularia oryzae* [142], *Trichophyton rubrum* [48], *Candida albicans* [143], and *Saccharomyces cerevisiae* [144]. *Pyricularia oryzae* exhibited 2109 succinylated sites distributed across 714 proteins [142]. Similarly, *Trichophyton rubrum* displayed 569 succinylated sites found on 284 proteins [48], while *Candida albicans* revealed 1550 succinylated sites occurring on 389 proteins [143]. Succinylation potentially serves a crucial function in the fundamental metabolic regulation of *Pyricularia oryzae*. Notably, more than 40 proteins associated with pathogenicity in *Pyricularia oryzae* were discovered to undergo succinylation. This observation suggests that succinylation is intricately linked to the pathogenicity of this organism [142]. Furthermore, *Trichophyton rubrum*, a prevalent dermatophyte species, relies on succinylated proteins to engage in a wide array of cellular operations. These include functions in translation, epigenetic control, and metabolic processes. Furthermore, the succinylation of 24 proteins associated with *Trichophyton rubrum*’s pathogenicity has been observed [48]. Likewise, in immunocompromised individuals, *Candida albicans*, one of the most prevalent human fungal pathogens, might rely on protein succinylation to crucially govern the TCA cycle. Hence, succinylation (Ksuc) could serve as a potential target for diminishing fungal pathogenicity [143].

The investigation into succinylation sites among various fungal species offers valuable insights on the intricate world of this post-translational modification. As we delve into the comparison of succinylation sites among various fungal species, it becomes evident that the regulatory role of succinylation extends beyond species-specific boundaries. Importantly, histone lysine, a central player in chromatin dynamics, is not exempt from such modifications. These modifications lead to charge shift as phosphorylation in histone residues, necessitating the use of highly precise mass spectrometry systems for their analysis [145]. In *Saccharomyces cerevisiae*, seven sites of lysine succinylation have been identified: H2AK13, H2AK21, H2BK34, H2BK46, H3K79, H4K31, and H4K77 [67]. The potential impact of these PTMs within the core domains of histones, closely engaged with DNA, suggests a role for succinylation in the modulation of histone–DNA interactions. To comprehend their functions, studies have utilized lysine substitutions, replacing them with arginine (R) or alanine (A) to prevent succinylation, or with glutamic acid (E) or aspartic acid (D) to simulate constant lysine succinylation [67]. The majority of these substitutions exhibited no discernible effect on the phenotype of *S. cerevisiae*. Notably, only the H4K77E mutant exhibited a loss of gene silencing at the telomeres and rDNA, implying a role of H4K77suc in stabilizing nucleosome assembly [146]

Similarly, in a study conducted by Frankovsky et al., performing proteomic mass spectrometry analysis of purified yeast mitochondria, revealed the presence of 314 succinylated mitochondrial proteins and identified 1763 succinylation sites. Among the structures impacted by succinylation is the mitochondrial nucleoid, a complex formed by mitochondrial DNA (mtDNA) and mitochondrial proteins. Their investigation unveiled that Abf2p, the primary constituent of mt-nucleoids responsible for compacting mtDNA in *S. cerevisiae*, undergoes succinylation in vivo at a minimum of thirteen lysine residues [144]. In a related study in *Aspergillus flavus*, an investigation into succinylated proteins, comparing strains with varying aflatoxin production capabilities, revealed 1240 lysine succinylation sites in 768 proteins. The *A. flavus* standard strain NRRL3357, subjected to sodium succinate treatment, was employed to scrutinize aflatoxin biosynthesis [58]. This exploration led to the pioneering discovery of Ksuc’s involvement in the secondary metabolic pathway and aflatoxin biosynthesis in *A. flavus* [56]. Employing tandem mass tag (TMT)-labeled quantitative lysine succinylome, they extensively documented the variations in Ksuc levels between *A. flavus* strains exhibiting high and low aflatoxin production under natural conditions. Subsequent functional analysis revealed a direct correlation between the upregulated proteins involved in carbon-related metabolism and aflatoxin production [147].

Meanwhile, Wang et al. performed a systematic analysis of the lysine succinylome in the model medicinal mushroom *Ganoderma lucidum*. Additionally, succinylated sites K90 and K106 were identified in the conserved Fve region of the immunomodulatory protein LZ8 [141], whereas in *pyricularia oryzae*, the analysis revealed a total of 2109 lysine succinylation sites within 714 proteins. Ten distinctive succinylation sequence patterns were recognized, with K*******Ksuc and K**Ksuc emerging as the two most favored ones. Lysine succinylation was observed in 10 crucial enzymes associated with the tricarboxylic acid (TCA) cycle. Among these, PGK and GAPDH exhibited the highest succinylation site count (12 sites), while FBP and PFK had the lowest (1 site) [142]. Likewise, the lysine succinylome from *Candida albicans* SC5314 was systematically characterized using an integrated proteomic approach. Notably, the comprehensive analysis revealed succinylation on every enzyme involved in the tricarboxylic acid (TCA) cycle. Aconitrate hydratase (ACO), a pivotal enzyme in the conversion of citric acid to isocitrate, has 19 succinylated sites [143]. Fachin et al. [49] reported mdr2-encoded ABC multidrug transporter has 2 succinylated sites, the AcuE-encoded malate synthase has 5 succinylated sites, and succinylated Rho GTPase Rho1 and Rho-GDP dissociation inhibitor (Rho-GDI), in dermatophytes infection [49].

In summary, the identification and comparison of these succinylation sites contribute to unraveling the complexities of cellular processes, shedding light on the functional roles and evolutionary aspects of this modification in fungal biology.

## 8. Tools Used to Discover Lysine Succinylated Proteins and Sites

### 8.1. Conventional Techniques Employed in the Identification of Lysine Succinylation

**Figure 5:**
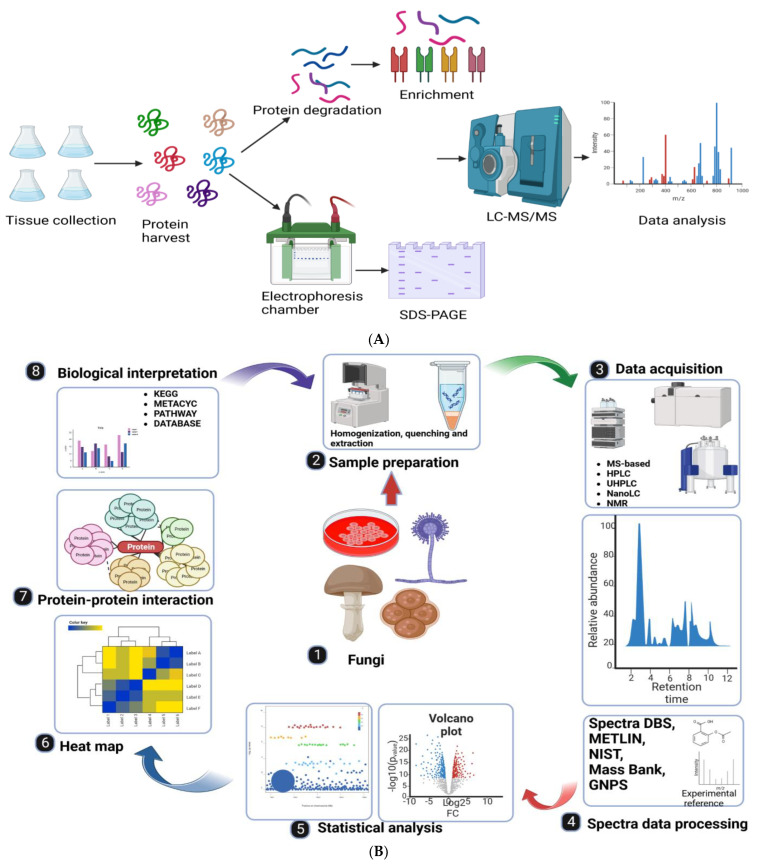
Process of identifying specific PTMs. (**A**,**B**) Initially, tissues are collected to extract total proteins. These proteins can then be enriched through various methods, such as immuno-affinity enrichment, or be separated using sodium dodecyl sulfate-polyacrylamide gel electrophoresis (SDS-PAGE). Following enrichment or separation, the peptides or proteins are analyzed using mass spectrometry, and the resulting data are subjected to further analysis. (**A**) Adapted with permission from [152]. Copyright © 2022, Yang, Tian and Keller. Figure created with BioRender.com.

Utilizing high-resolution liquid chromatography-tandem mass spectrometry (LC-MS/MS) and sensitive immune-affinity purification, a proteomic approach was employed to identify previously unreported Ksuc sites and succinylated proteins that constitute the overall succinylome. This method has led to the discovery of novel insights into lysine succinylation [148]. Afterward, Nano-LCMS/MS [56,58,149] and high-performance liquid chromatography-tandem mass spectrometry (HPLCMS/MS) [150] were employed to investigate the succinylome. The utilization of bioinformatics analysis holds a pivotal role in comprehending the potential functions linked to succinylation. To elucidate the potential roles of succinylation, researchers have employed diverse tools such as the Gene Ontology (GO) annotation, Kyoto Encyclopedia of Genes and Genomes (KEGG), and the Protein–Protein Interactions (PPIs) network sourced from the STRING database, as well as Cytoscape analysis [151]. Succinylome mapping serves as a valuable tool for uncovering regulatory networks and novel characteristics of succinylation. These approaches are facilitating the exploration of lysine succinylation (Ksuc) in a growing range of organisms such as *Aspergillus flavus* and other fungi (Figure 5).

### 8.2. Computational Tools to Predict Lysine Succinylation Sites

Conventional approaches utilized to detect Ksuc are rooted in proteomic methodologies, like LC-MS/MS [148,149,150]. However, these methods are intricate, costly, and demand considerable time [153]. Consequently, several online tools have been devised to forecast potential Ksuc sites. In 2015, a pioneering effort by Zhao et al. [154] introduced the initial succinyl site prediction tool, SucPred. This tool employed a specialized type of semi-supervised machine learning known as the positive samples-only learning (PSoL) algorithm to train the model [154]. In the same year, Xu and colleagues utilized the support vector machine (SVM) along with an incorporated peptide position-specific propensity, which was transformed into a generalized form of pseudo-amino acids (PseAAC) composition. This innovative approach led to the development of a novel prediction tool named iSuc-PseAAC [155]. Furthermore, these researchers went on to establish SuccFind using a feature selection algorithm that encompassed an amino acid component (AAC), the composition of k-spaced amino acid pairs (CKSAAP), and the amino acid index (AA index) [156]. In a similar vein, Jia et al. [157,158] created two prediction tools, namely pSuc-Lys and iSuc-PseOpt, leveraging a PseAAC descriptor through a random forest RF classifier [157,158].

In 2017 and 2018, López et al. [159,160] developed the Success and SucStruct predictors, which hinged on the amalgamation of secondary structure feature (SF) SSpre with a bigram and decision tree (DT) algorithm. These innovations exhibited robust sensitivity, accuracy, and a favorable Matthew’s correlation coefficient, thereby showcasing their effectiveness [159,160]. Likewise, Dehzangi et al. developed two prediction tools, namely SSEvol-Suc and PSSM-Suc, employing evolutionary features, sequence-based attributes, and a DT classifier [161,162]. Utilizing SVM and an ensemble learning algorithm, the PSuccE tool was trained to forecast protein succinylation sites [163]. Similarly, pSuc-PseRat and iPTM-mLys employed the utilization of ratios of sequence coupling and forest classifiers to predict protein succinylation sites [164,165]. Furthermore, SuccinSite, GPSuc, and SuccinSite2.0 were formulated by Hasan et al. These tools harnessed amino acid patterns and properties, as well as multisequence features, in conjunction with RF classifiers [166,167]. Both SuccinSite2.0 and GPSuc integrate various species classifications in their frameworks [167,168,169].

In 2019, CNN-SuccSite was introduced by Huang et al., offering the capability to investigate the specificity of substrate sites for succinylation [170]. In recent times, Inspector was introduced by Zhu et al., utilizing a random forest algorithm along with feature encoding and sequence-based schemes. This tool exhibits a notable level of accuracy in predictive modeling [171]. Through the implementation of a deep learning-based approach and embedding techniques, DeepSuccinylSite was created by Thapa et al. This method aims to identify succinylation sites within protein primary structures [172]. Ning et al. devised an innovative approach to predict succinylation sites with their tool SSKM_Succ, which combines K-means clustering with a unique semi-supervised learning algorithm [173]. Following that, a hybrid learning framework called HybridSucc was introduced by Ning and his team. This framework merges conventional machine-learning algorithms and deep-learning models into a unified architecture [174]. In 2020, an IFSLightGBM (BO)-based predictive model was established by Zhang et al. [175]. This model introduced the iterative feature selection (IFS) method along with various feature selection techniques, and it was coupled with the LightGBM classifier to effectively minimize noise and extraneous information. The FS-LightGBM (BO) model demonstrated notable performance, achieving an accuracy of 0.7392, a Matthews correlation coefficient of 0.4771, and an impressive F-measure of 0.7255 [175].

The HybridSucc database presently documents 8710 identified proteins that have undergone succinylation, featuring a total of 23,866 Ksuc sites across 13 distinct species [172]. The prediction of succinylation sites was further enhanced with the introduction of MDCAN-Lys [176] and LSTMCNNsucc [175] tools. LSTMCNNsucc, in particular, innovatively merges a convolutional neural network (CNN) and long short-term memory (LSTM), forming a deep learning model that excels in the accurate anticipation of succinylation sites [177]. It is important to acknowledge that succinylation frequently coincides with other PTMs, such as acetylation. Tools like predML-Site and ‘iMul-kSite’ have been developed to identify these overlapping PTMs [68,178]. Predictive tools capable of identifying the interaction between Ksuc and other PTMs contribute to uncovering intricate regulatory networks within cells. These resources offer a user-friendly avenue for researchers lacking a background in bioinformatics to uncover novel Ksuc sites and proteins.

## 9. Conclusions and Future Prospects

In conclusion, this review has provided a comprehensive overview of the impact of lysine succinylation on the biology of fungi. The key findings and outcomes of this review can be summarized as follows: the specific residues and proteins impacted by this post-translational modification have been clarified through a thorough investigation of lysine succinylation sites in fungal proteins. The physiology, metabolism, and virulence of fungi have been explained in relation to the functional consequences of lysine succinylation. Among the important biological functions played by lysine succinylation are those of energy metabolism, stress reactions, and gene transcription. Different fungal species have different patterns of lysine succinylation, which has been highlighted in this review. This highlights the need for species-specific research to fully comprehend the regulatory role of this modification.

Even though a lot has been accomplished, there are still a few research directions that should be pursued in the future. Validating the functional impact of lysine succinylation on particular fungal proteins will require more experimental research. This could entail phenotypic analysis of fungi with modified succinylation patterns and genetic modification. In addition, it is important to perform extensive proteomic analyses in order to map lysine succinylation events throughout a variety of fungal species. This will improve our comprehension of the divergence and evolutionary conservation of succinylation in fungi. Furthermore, lysine succinylation in fungi influences regulatory networks, and integrating systems biology techniques like network analysis and computational modeling can offer a comprehensive picture of these networks. Meanwhile, further research should focus on developing antifungal agents that target enzymes involved in succinylation or de-succinylation processes, as there is a possibility that lysine succinylation affects fungal virulence.

## Figures and Tables

**Table 1:** Lysine succinylated enzymes in fungi.

Fungus	Enzymes	Function	References
*Trichophyton rubrum*	There are 18 proteins annotated as acetyltransferases (TERG_00136T0, TERG_00160T0, TERG_00920T0, TERG_00960T0, TERG_02546T0, TERG_03442T0, TERG_03711T0, TERG_04055T0, TERG_04687T0, TERG_04983T0, TERG_05174T0, TERG_05450T0, TERG_05561T0, TERG_06411T0, TERG_06572T0, TERG_07217T0, TERG_07375T0, and TERG_07548T0	Additional experiments are required to investigate whether one or several of these acetyltransferases possess succinyltransferase activities, or if there is another succinyltransferase in *T. rubrum*.	[48]
*Yeast*	In yeast, five Sir2 proteins have been acknowledged (Sir2p and Hst1–4)	Can catalyze other forms of lysine acylation. In yeast, Hst2 exhibits a higher affinity for binding propionyl-lysine and butyryl-lysine compared to acetyl-lysine	[117,118,119]
*Trichophyton rubrum*	*T. rubrum*, five potential deacylases have been designated as Sir2 family histone deacetylases (TERG_03010T0, TERG_03268T0, TERG_05234T0, TERG_06970T0, and TERG_07330T0).	Further investigations are required to explore the activities of these Sir2 proteins. They are curious about whether one of these deacetylases might be involved in desuccinylase activities or if there is another desuccinylase present in these dermatophytes	[48]

## Data Availability

No new data were created.
